# Hypertension Associated with Coarctation of the Aorta Revisited: Case-Based Update from Experience of Three Children

**DOI:** 10.1155/2013/716438

**Published:** 2013-09-05

**Authors:** Ali Baykan, Mustafa Argun, Abdullah Özyurt, Özge Pamukçu, Kazım Üzüm, Nazmi Narın

**Affiliations:** Division of Pediatric Cardiology, Department of Pediatrics, Erciyes University Medical Faculty, Melikgazi, 38039 Kayseri, Turkey

## Abstract

Coarctation of the aorta (CoA) can present with different clinical pictures depending on the severity of the narrowness in the coarcted aortic segment in an age range between newborn and adolescence. Sometimes, it can cause intracranial hemorrhage or infarction when diagnosis and treatment are delayed. The aim of this report is taking attention to CoA as a cause of systemic hypertension and is also emphasizing the differences of diagnostic approach for hypertension in children from adults. Two cases of hypertensive cerebral hemorrhage and one case of hypertensive cerebellar infarction associated with CoA are reported. These cases help us to pay attention to the possibility of CoA in adolescents with hypertensive stroke. We want to emphasize the importance of physical examination for evaluation of hypertension and to impress the diagnostic approach for secondary hypertension in children.

## 1. Introduction

 Coarctation of the aorta (CoA) is typically a discrete stenosis of the proximal thoracic aorta; CoA is an uncommon cause of cerebral hemorrhage and cerebellar infarction in children [1]. The diagnosis of CoA may be missed in children with cerebral hemorrhage if there is no high degree of suspicion. We report two adolescents who were presented with acute cerebral hemorrhage and one child who was presented with acute cerebellar infarction, which was the result of untreated hypertension/CoA. The aim of this report is underlining the importance of early diagnose and management of CoA as a cause of systemic hypertension and also emphasizing the differences of diagnostic approach and treatment for hypertension in children from adults. We would like to share our experience about three cases of hypertension associated with CoA.

*Case 1.* A 15-year-old male developed sudden onset of loss of consciousness, vomiting, and seizure. We learned that the patient has used angiotensin-converting enzyme inhibitor for hypertension since the last 5 years. Significant clinical findings were poor general status, loss of consciousness, severe hypertension, I/VI systolic ejection murmur on left upper sternal border, and absent femoral pulses. She was managed at pediatric intensive care unit and required mechanic ventilation.

**Figure 1:**
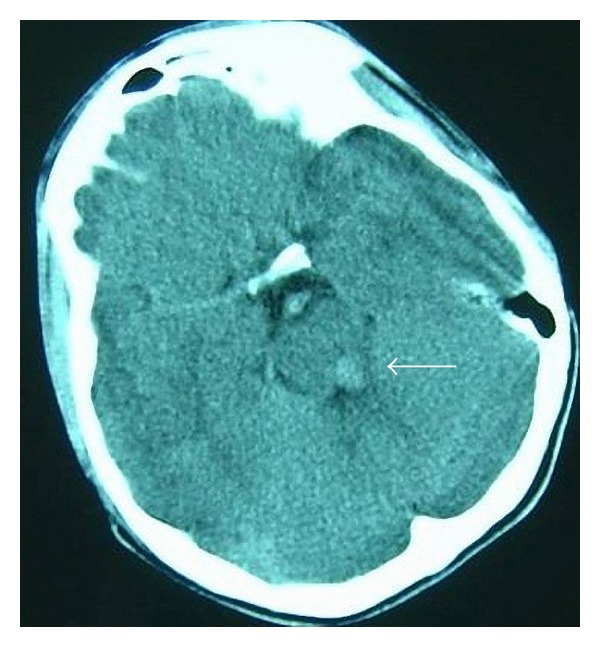
Brain CT scan demonstrating hyperdense appearance consistent with hemorrhage within the pons.

Echocardiography revealed severe left ventricular hypertrophy and CoA. There was 90 mm Hg pressure gradient by Doppler echocardiography at the stenotic segment. The cranial computed tomography (CT) scan showed hyperdense appearance within the pons consistent with hemorrhage (Figure 1). At cardiac catheterization, there was 80 mm Hg pressure gradient at the level of CoA. Cerebral angiogram demonstrated severe vasospasm at basilar artery without microaneurysm. Balloon angioplasty was performed to CoA. After the procedure, pressure gradient at CoA decreased to 35 mm Hg. But patient died within a couple of days because of respiratory distress related to hemorrhage at pons.

*Case 2. *A 14-year-old previously healthy male admitted to emergency service with sudden loss of consciousness, seizure, and headache. The patient was hospitalized to neurosurgery intensive care unit for cerebral stroke. On physical examination, he had a blood pressure of 220/100 mm Hg (right arm). The blood pressure in the right and left arms was similar and higher than lower limbs (50 mm Hg). On auscultation, II/VI systolic ejection murmur was heard, and weak femoral pulses were noted. There was left hemiplegia and facial palsy on neurologic examination.

 The electrocardiogram revealed irregular rhythm with normal QRS axis, sinus pause, and left ventricular hypertrophy. A posteroanterior chest X-ray demonstrated cardiomegaly, laterally displaced apex, and rib notching. On echocardiography, there were severe left ventricular hypertrophy with preserved systolic function and a 90 mm Hg Doppler gradient across the site of CoA. The CT scan of the cranial demonstrated a large hemorrhage within the right cerebral hemisphere. One year after stroke, the CT scan of head showed brain white matter volume reduction and encephalomalacic changes. At cardiac catheterization, there was 97 mm Hg pressure gradient across CoA site. The balloon angioplasty was performed to the CoA. After the procedure, aortic diameter improved significantly, and pressure gradient decreased to 34 mm Hg at CoA site.

*Case 3. *An 8-year-old female with no history of hypertension presented to the emergency department with syncope associated with exercise. Blood pressure was 140/90 mm Hg on both arms. Cardiac auscultation revealed II/VI systolic murmur along the left sternal border. Weak femoral pulses were noted. 

**Figure 2:**
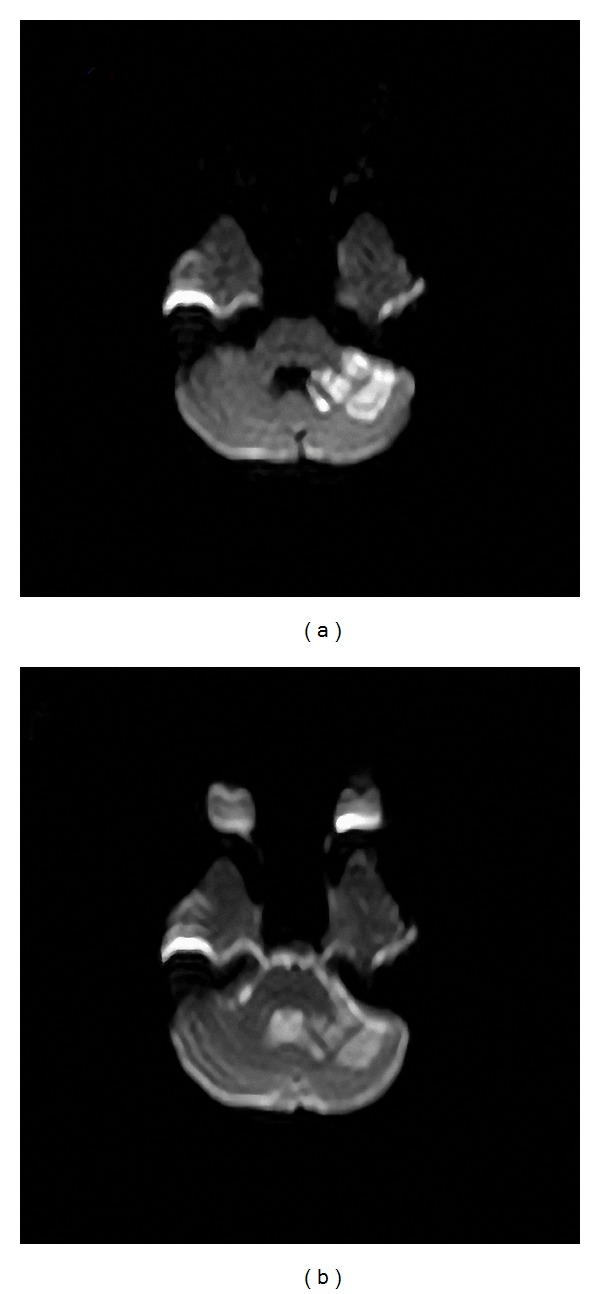
Brain diffusion MRI showing acute cerebellar infarction on the left cerebellar hemisphere.

**Figure 3:**
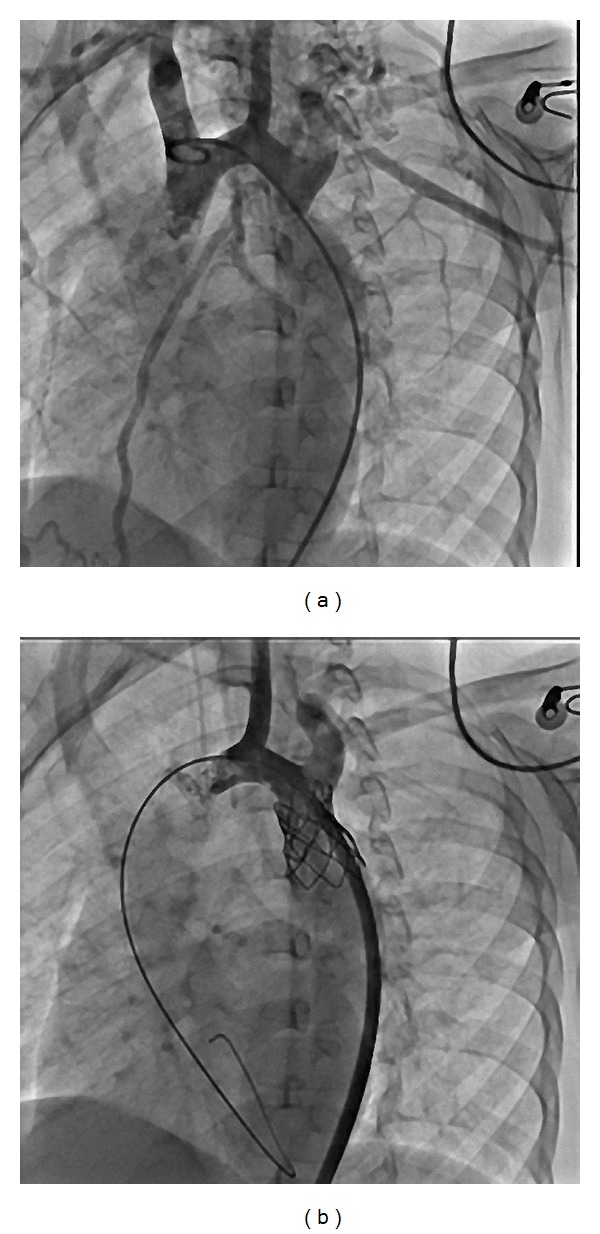
(a) Descending aortogram showing severe discrete coarctation of the aorta, (b) dilated coarctation after implantation of a 28 mm stent.

An echocardiography showed moderate left ventricular hypertrophy, bicuspid aortic valve, and isthmus aorta with discrete narrowing. Doppler color flow study found 35 mm Hg gradient across the site of the CoA. Magnetic resonance imaging revealed left cerebellar infarction (Figure 2). Aortography showed severe discrete coarctation. After implantation of a 28 mm diameter stent, systolic pressure gradient during the procedure decreased from 30 mm Hg to 5 mm Hg (Figure 3). Unfortunately, one month later, follow-up physical examination revealed cerebellar ataxia, and slight motor impairment of the left hand after the stroke had been enrolled to physical rehabilitation program.

## 2. Discussion

Hypertension, elevation of blood pressure within the arteries, arises from a perturbation of the usual balance between left ventricular contraction, arterial wall elasticity, blood volume, and blood viscosity [2]. Normative blood pressure tables are stratified by age, gender, and height. Accordingly, normotensive; mean systolic BP (SBP) and diastolic BP (DBP) <90th percentile for age, height, and sex at first screening; prehypertensive: mean SBP or DBP ≥90th percentile but <95th percentile at first screening or mean SBP or DBP initially ≥95th percentile but <95th percentile at either second or third screening; Stage 1 hypertensive: mean SBP or DBP ≥95th percentile for all three screenings with BP ≥95th percentile but ≤99th percentile +5 mm Hg on ≥2 screening sessions; and Stage 2 hypertensive: mean SBP or DBP ≥95th percentile for all 3 screenings with BP >99th percentile +5 mm Hg on ≥2 screening sessions [3]. 

Hypertension affects <1% of infant and young children. As a result of the systemic hypertension, stroke may occur if it is left undetected and untreated [4]. When hypertension develops as a consequence to another disease, it is called secondary hypertension. The most common cause of hypertension varies with age. The etiologic factors of secondary hypertension in children mostly include renal-vascular diseases, cardiovascular factors, and endocrine problems. When a secondary cause leading to hypertension cannot be found, it is called primary or essential hypertension. Numerous factors including heredity, obesity, diet, and stress play a role in the development of essential hypertension. Essential hypertension is very rare in children [2]. Causes of hypertension are shown in the following list. Children and adolescents with essential hypertension are usually asymptomatic and elevation in blood pressure is usually mild. On the other hand, when there is an underlying secondary cause, increase in blood pressure is usually more severe. 

*Causes of Hypertension*

- Secondary hypertension
  - Renal diseases:
    - chronic pyelonephritis,
    - glomerulonephritis,
    - congenital dysplastic kidney,
    - polycystic kidney disease,
    - vesicoureteral reflux nephropathy.
  - Vascular diseases:
    - coarctation of the aorta,
    - umbilical artery catheterization with thrombus formation,
    - renal artery stenosis,
    - renal vein thrombosis.
  - Endocrine diseases:
    - cushing syndrome,
    - hyperaldosteronism,
    - pheochromocytoma,
    - diabetic nephropathy.
  - Psychological causes:
    - mental stress,
    - anxiety.
  - Pharmacologic causes:
    - exogenous steroids,
    - oral contraceptives,
    - decongestants,
    - stimulants used to treat attention-deficit hyperactivity disorder.
- Essential hypertension.

A careful history and information obtained from a complete physical examination will preclude unnecessary and frequent costly laboratory workup and imaging studies. Physical examination findings of the underlying disease suggest the following: abdominal mass; polycystic renal disease, acne, and increased hairiness; Cushing's syndrome, excessive sweating, tachycardia, and flushing; pheochromocytoma and retarded growth; chronic renal failure, arthritis, and malar rash; systemic lupus erythematosus, murmur, and decreased circulation of the lower extremities; CoA [4]. Physical examination should definitely include palpation of all extremity pulses as well as measurement of both upper extremity and lower extremity pressures. In children, pressure of the upper extremities is normally 10–20 mm Hg less than that of lower extremities. In a hypertensive child, screening tests at the time of diagnosis should include basic serum biochemistry, complete blood count, urinary analysis and culture, renal ultrasonography, echocardiography, and ophthalmic examination. 

Deal et al. have reported 110 cases with severely raised blood pressure which deemed to require emergency management. Underlying secondary causes have been shown in 90% of cases. CoA has been found in four patients [5]. CoA which is one of the rare causes of severe hypertension accounts for approximately 6% to 8% of all congenital heart diseases. The anomaly occurs twice often in males than females. CoA is associated with a bicuspid aortic valve in 20% to 85% of patients. Coarctation of aorta can present with different clinical pictures depending on the severity of the narrowness in the coarcted segment in an age range between newborn and adolescence and more. Critical coarctation in neonates can present with shock, absent femoral pulse, or murmur, whereas during infancy and childhood the usual presentation includes weak femoral pulse, cardiac murmur, or heart failure. In adolescents it can appear as a cause of systemic hypertension apart from the mentioned examination findings. Sometimes, it can cause intracranial hemorrhage or infarction when diagnosis and treatment are delayed [6]. CoA always must be considered in the differential diagnosis because blood pressure control cannot be succeeded with antihypertensive agents alone. Hypertension is usually found in vessels proximal to the obstruction site in patients with CoA. The hypertension is not due to the obstruction alone but also includes neurohumoral mechanisms in CoA. Surgical or transcatheter interventions have to be performed for treatment of hypertension at CoA [7].

As a result of the systemic hypertension, cerebral/cerebellar hemorrhage and infarction may occur if it is left undetected and untreated. In our first case, hypertension has been determined 5 years ago. Since this time only antihypertensive agents were given, but the etiologic studies of hypertension even physical examination had not been performed carefully. In our second and third case, hypertension had not been detected until the patient has a stroke. In our opinion, physicians are not only responsible for treatment but also responsible for preventive medical care of such these patients. So physical examination of an ill child admitted for any cause must be performed carefully. 

 The most common risk factors for any type of intracranial hemorrhage in children are intracranial vascular anomalies, congenital heart disease, brain tumors, sepsis, thrombocytopenia, and coagulation factor deficiencies. Subarachnoid or intracerebral hemorrhage is the result of ruptured aneurysm or normal vessel [6]. In our first case, cerebral angiogram did not show any microaneurysm. Cerebral CT scan revealed bleeding in the pons. Clinical presentation of cerebral hemorrhage in children varies with age. In children younger than 6 years the clinical presentations are often nonspecific, such as mental changes, seizures, and vomiting. In children 6 years and older, headache, mental changes, focal neurological deficits, and vomiting are the most common clinical signs [8]. In line with the literature, our cases had sudden loss of consciousness, vomiting, and seizure. 

Our asymptomatic hypertensive second case was presented with cerebral hemorrhage, but the first case has been diagnosed with essential hypertension and was not evaluated for the etiology by another center and they tried to give angiotensin converting enzyme inhibitors. Although essential hypertension is frequent in adults, it is very rare in childhood. Besides detailed history, complete physical examination, basic laboratory workup further tests should be done in all newly diagnosed hypertensive pediatric patients. 

## 3. Conclusion

We report three children with stroke in association with undetected and untreated CoA. Occult aortic coarctation should be suspected in young hypertensive patients, especially in those with cerebrovascular syndromes. Coarctation of the aorta can be strongly suspected on the basis of careful physical examination. Blood pressure measuring is and must be the part of routine physical examination in children three years of age and older. If a pediatric patient has hypertension, further examination must be done for etiology, and causes of secondary hypertension must be evaluated.
